# Prescriber practices and patient adherence to artemisinin-based combination therapy for the treatment of uncomplicated malaria in Guinea, 2016

**DOI:** 10.1186/s12936-019-2664-7

**Published:** 2019-01-25

**Authors:** Alioune Camara, Leah F. Moriarty, Timothée Guilavogui, Papa Sambou Diakité, Joseph Souba Zoumanigui, Sidikiba Sidibé, Ibrahima Bah, Ibrahima Kaba, Djebory Kourouma, Koho Zoumanigui, Mateusz Plucinski

**Affiliations:** 1 National Malaria Control Programme, Conakry, Guinea; 2 Department of Public Health, University of Conakry, Conakry, Guinea; 3 Division of Parasitic Diseases and Malaria, Center for Global Health, Centers for Disease Control and Prevention, US President’s Malaria Initiative, Atlanta, GA USA; 4 Catholic Relief Services, Conakry, Guinea

**Keywords:** Malaria, Adherence, Drug intake, Lumefantrine, Amodiaquine, Guinea, ACT

## Abstract

**Background:**

The World Health Organization recommends the use of artemisinin-based combination therapy (ACT) to treat uncomplicated malaria for the control of malaria across the world. There are several types of ACT used across malaria-endemic countries, yet there is little information about preferences and adherence practices regarding different types of ACT. The objective of this study was to evaluate levels of adherence to two types of ACT, artemether–lumefantrine (AL) and artesunate + amodiaquine (ASAQ), for the treatment of uncomplicated malaria among prescribers and patients in Guinea in 2016.

**Methods:**

The study included a review of records of malaria patients and three health-facility, cross-sectional surveys. Patients diagnosed with uncomplicated malaria and prescribed ACT (n = 1830) were recruited and visited in their home after receiving the medication and administered a questionnaire regarding ACT adherence. Prescribers (n = 115) and drug dispensers (n = 43) were recruited at the same public health facilities and administered questionnaires regarding prescribing practices and opinions regarding the national treatment policies and protocols.

**Results:**

According to the registry review, 35.8% of all-cause consultations were recorded as malaria. Of these, 26.6% were diagnosed clinically without documentation of laboratory confirmation. The diagnosis of uncomplicated malaria represented 64.1% of malaria cases among children under 5 years and 74.9% of those 5 years of age and older. An ACT was prescribed for 83.5% of cases of uncomplicated malaria. Among participants in the study, ACT adherence was 95.4% (95% CI 94.4, 96.3). Overall, about one in four patients (23.4%; 95% CI 21.5, 25.3) reported experiencing adverse events. While patients prescribed ASAQ were significantly more likely to report experiencing adverse effects than patients on AL (p < 0.001), given the overall high adherence, there was no evidence of a statistically significant difference in adherence between AL and ASAQ. Patients 5 years or older who reported experiencing adverse events were more likely to be non-adherent.

**Conclusion:**

Although there were more reported adverse events associated with ASAQ when compared with AL, both prescribers and patients were found to be mostly adherent to ACT for the treatment of malaria, regardless of ACT type.

**Electronic supplementary material:**

The online version of this article (10.1186/s12936-019-2664-7) contains supplementary material, which is available to authorized users.

## Background

Following the discovery of elevated chloroquine and sulfadoxine–pyrimethamine (SP) resistance, in the early 2000s the World Health Organization (WHO) proposed a new strategy for malaria control for all malaria-affected countries across the world. This strategy recommends the use of artemisinin-based combination therapy (ACT) for the treatment of uncomplicated malaria [1, 2]. In sub-Saharan Africa, the two most frequently recommended ACT is artesunate/amodiaquine (ASAQ) and artemether/lumefantrine (AL) [1]. Additionally, to prevent empiric treatment of fever without laboratory confirmation for malaria, national malaria control programmes (NMCP) introduced the use of rapid diagnostic tests (RDT) at health facilities. This ensures that an ACT is only prescribed for confirmed cases of uncomplicated malaria [3].

Malaria is a major public health problem in Guinea, endemic for malaria in its entirety. In the most recent household survey in Guinea in 2016, 15% of children under 5 years old in the community had slide-detectable parasitaemia [4]. Routinely collected health facility data show that 31% of all-cause outpatient consultations are due to malaria [5].

Like other malaria-endemic countries in Africa, Guinea adopted a treatment policy for uncomplicated malaria based on ACT in 2005 [6]. Based on this national malaria control policy, all confirmed cases of uncomplicated falciparum malaria must be treated with an oral ACT. The main driver of this policy was the approval of ACT by WHO. Guinea NMCP chose two types of ACT based on efficacy, cost, tolerance, and market availability: ASAQ and AL. In practice, ASAQ was the primary ACT procured for Guinea. However, starting in 2015, AL began to be procured in more substantial quantities, originally to replace ASAQ as the first line treatment in areas where seasonal malaria chemoproprevention (SMC) using SP + AQ began. ASAQ is not recommended to use as a first line drug in areas where SMC is being implemented [7]. However, anecdotal reports of strong patient preference for AL in areas where both ACT were available motivated the NMCP to commission a study to obtain more data on ACT adherence in Guinea.

The effectiveness of ACT is not guaranteed without an accurate prescription that is properly taken by the patient. The lack of adherence to an ACT can contribute to the development of anti-malarial resistance, severe malaria, and in some cases death. It is for these reasons that some in the scientific community have cited the understanding of determinants of ACT adherence as among the most urgent questions for case management [8].

A number of studies have evaluated patient adherence to anti-malarial drugs and associated factors. The documented level of patient adherence to ASAQ varies from 48% in Sierra Leone [9] to 75% in the Democratic Republic of Congo [10], to 93% in Ghana [11]. One qualitative study comparing ACT adherence in northern Ghana found that patients preferred AL over ASAQ due to adverse events experienced with ASAQ [12]. The level of patient adherence to AL varied from 38.7% in Ethiopia in 2011 [13], to 90.5% in Nigeria in 2013 [14, 15]. One review found that demographic factors, such as gender, socio-economic status, or age were not found to be significantly or systematically associated with adherence [15].

There are few published studies on the subject of ACT adherence in Guinea. This paper describes the results of a study completed in 2016. The overall objective of the study was to evaluate the level of adherence of prescribers and users of ACT for the treatment of uncomplicated malaria in Guinea in 2016. Specifically, the study sought to evaluate the level of adherence to ACT prescription for uncomplicated malaria among prescribers, ACT adherence among patients with uncomplicated malaria, the frequency of ACT prescription to treat uncomplicated malaria, and the factors associated with non-adherence to ACT. The objectives of the study were in the context of public health facilities in Guinea.

## Methods

### Study design and period

There were three components of the described study. The first was a retrospective review of health facility registers to determine the frequency of ACT prescription that covered the period 1 May to 31 July, 2016. Additionally, two cross-sectional surveys were administered between 1 August and 30 October, 2016. The first targeted patients prescribed ACT in a sample of public health facilities; the second targeted prescribers and drug dispensers in the same health facilities.

### Study area

**Fig. 1:**
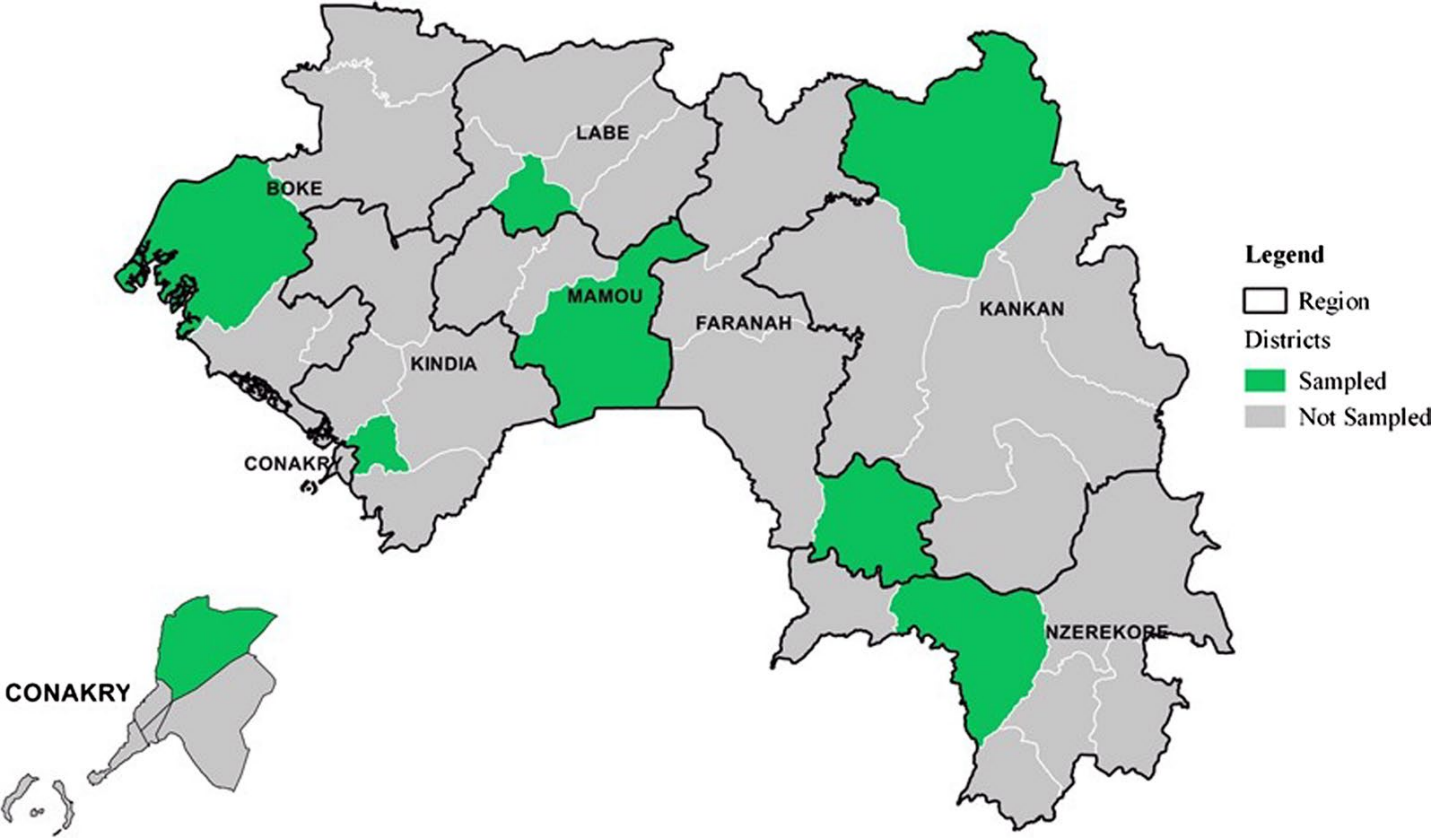
Map of sampled districts for surveys and registry reviews, 2016, Guinea

The study was carried out in the eight administrative regions of Guinea: Boké, Kindia, Labé, Mamou, Faranah, Kankan, N’zérekoré, and Conakry, at public health facilities (regional hospitals, district reference hospitals, urban and rural health centres, regional health centres, community medical centres). In hospitals, services included were paediatric, medical emergency, general medicine, maternity, and pharmacy units. In urban and rural health centres, the primary curative consultation and dispensary units were included (Fig. 1).

### Study population

All patients diagnosed with uncomplicated malaria during the study period and prescribed ACT and agreed to participate in the survey in sampled health facilities across the eight regions of Guinea were included in the study. Patients interviewed on the first day who were not seen after ACT treatment began were excluded from the analysis. Prescribers and drug dispensers of ACT who worked at the sampled health facilities across the eight regions of Guinea and agreed to participate in the surveys were also included.

### Sampling

A two-stage, probability sampling procedure was used that included all eight administrative regions. At the first stage, one random health district was chosen from each of the eight administrative regions. The health districts chosen were Boké, Coyah, Kissidougou, Labé, Macenta, Mamou, Siguiri, and the commune of Ratoma in Conakry. At the second stage, in each district, a random sample of an urban health centre, a rural health centre, and regional or district reference hospital was taken (Additional file 1: Table S1), for a total of 24 health facilities. All prescribers/drug dispensers of ACT in the facilities visited were invited to be interviewed. A minimum of one healthcare provider and one drug dispenser was included from each health facility. For patients, the sample size was calculated based on a non-adherence level of 25% ± 10% and 95% confidence intervals [15]. Adding 20% of the sample to take into account loss to follow up, the minimum sample size of 175 patients for each region was determined based on results of a field test of the survey.

### Data collection

Health centre registers were used to fill out the template for the retrospective analysis. The template included information about testing, diagnostics and treatment of malaria in the sampled health facilities in the 3 months before the survey (May–July). The treatment of uncomplicated malaria was divided by ACT (ASAQ or AL), ACT not specified, commercial ACT (unsubsidized commercial products, such as Bimalaril), non-ACT (quinine, amodiaquine), and not indicated. Patients were recruited at the exit of their consultations to be included and interviewed consecutively. Prescribers at included health facilities participated in informing patients about participation in the study and asked that patients save the blister pack of the ACT at the end of the regimen, without telling them why.

On the day of recruitment (day 0), basic sociodemographic information, including address (physical address, telephone number), and prescriptions as written in the logbooks were collected. Patients were revisited at their home or in the health facility on the day after the last day of the ACT (4th day or after), and a small proportion (about 10%) were interviewed by phone. During this visit, study interviewers verified the availability of and inspected the ACT blister pack to see how many tablets were left, if any. Additionally, information regarding taking the medication, including reasons for missing any doses and any adverse events were collected. Patients who were not available at their home on the fourth day were revisited the four following days, and if not seen were considered lost to follow up.

In each sampled health facility, consenting prescribers and drug dispensers were recruited at the end of the work day to avoid disruption of their day. Separate questionnaires were administered for prescribers and drug dispensers of ACT that included information regarding knowledge and attitudes about the prescription and dispensing of ACT and on the level of adherence to the prescription of ACT for uncomplicated malaria, (total agreement was considered adherent, disagreement or partial was considered non-adherent) (Additional file 2). All the data collection tools were field tested in three health facilities in the Matam commune of Conakry and not included in the data for the study. All personal identifiable information such as physical address and telephone numbers was stored in a password-protected database.

### Data analysis

Double data entry of interview responses was performed. Each interviewer had his or her own laptop. All data were re-entered after interviewer teams completed surveys and reconvened after data collection. Any discrepancies between the two data entries were reconciled based on the original paper forms. Data entry and coding for the questionnaire were entered into Microsoft Excel using EpiData (Version 3.1, EpiData association, Odense, Denmark). The analysis was done using SPSS (version 24, IBM Corp, USA). Descriptive analyses included the calculation of proportions for categorical variables, medians, means, and standard deviations for continuous variables.

Patients were classified into two adherence-related categories: adherent (those who stated that they took all doses in the pack with or without the blister pack within 3 days of being prescribed), and non-adherent (those who stated that they did not complete the treatment or those who still had tablets left in the blister pack). The ‘adherent’ category was used as a reference in logistic regression models. Simple and multivariate logistic regression models were developed to determine factors associated with being non-adherent. A stepwise, forward selection approach was used to build a multivariate logistic regression model, including p < 0.20 as a cut-off for inclusion of each variable. Only variables statistically significant at the 5% level were kept in the final model. Odds ratios and confidence intervals were calculated for the final model. Statistical tests were considered significant at p < 0.05.

### Ethical considerations

Verbal agreement was obtained from all subjects included in the study, including consent from a parent or caregiver for patients under the age of 18 years. Interview sheets did not include identifiable information of those interviewed and were kept in a secure location. The protocol was reviewed and classified as a non-research programme evaluation by the CDC Center for Global Health Office of the Associate Director for Science (2018-073).

## Results

### Retrospective review

In the 3 months preceding the survey, 32,899 all-cause consultations of which 11,786 (35.8%) were malaria (including uncomplicated and severe) were documented in the registers of the 24 health facilities. Among the malaria cases recorded, 4591 (39%) were under 5 years of age (Table 1).

**Table 1:** Quality of malaria diagnosis by age and region, evaluated by a retrospective review of health facility registers from 1 May to 31 July, 2016, Guinea (N = 11,786)

	Boké	Kindia	Faranah	Labé	Nzérékoré	Mamou	Conakry	Kankan	Total
	n (%)	n (%)	n (%)	n (%)	n (%)	n (%)	n (%)	n (%)	n (%)
< 5 years	**n = 314**	**n = 737**	**n = 924**	**n = 312**	**n = 612**	**n = 938**	**n = 290**	**n = 464**	**n = 4591**
RDT	170 (54.1)	349 (47.4)	560 (60.6)	280 (89.7)	273 (44.6)	529 (56.4)	189 (65.2)	16 (3.4)	2366 (51.5)
Microscopy	121 (38.5)	241 (32.7)	213 (23.1)	8 (2.6)	318 (52.0)	31 (3.3)	81 (27.9)	45 (9.7)	1058 (23.0)
No laboratory confirmation	23 (7.3)	147 (19.9)	151 (16.3)	24 (7.7)	21 (3.4)	378 (40.3)	20 (6.9)	403 (86.9)	1167 (25.4)
Diagnosis of uncomplicated malaria	245 (78.0)	623 (84.5)	397 (43.0)	153 (49.0)	350 (57.2)	623 (66.4)	271 (93.4)	282 (60.8)	2944 (64.1)
Diagnosis of severe malaria	69 (22.0)	114 (15.5)	527 (57.0)	159 (51.0)	262 (42.8)	315 (33.6)	19 (6.6)	182 (39.2)	1647 (35.9)
≥ 5 years	**n = 561**	**n = 706**	**n = 858**	**n = 898**	**n = 1047**	**n = 1382**	**n = 1219**	**n = 524**	**n = 7195**
RDT	222 (39.6)	386 (54.7)	321 (37.4)	536 (59.7)	692 (66.1)	674 (48.8)	556 (45.6)	22 (4.2)	3409 (47.4)
Microscopy	296 (52.8)	246 (34.8)	167 (19.5)	1 (0.1)	307 (29.3)	113 (8.2)	578 (47.4)	116 (22.1)	1824 (25.4)
No laboratory confirmation	43 (7.7)	74 (10.5)	370 (43.1)	361 (40.2)	48 (4.6)	595 (43.1)	85 (7.0)	386 (73.7)	1962 (27.3)
Diagnosis of uncomplicated malaria	506 (90.2)	600 (85.5)	573 (66.8)	589 (65.6)	904 (86.3)	1174 (84.9)	1122 (92.0)	412 (78.6)	5880 (81.7)
Diagnosis of severe malaria	55 (9.8)	106 (15.0)	285 (33.2)	309 (34.4)	143 (13.7)	208 (15.1)	97 (8.0)	112 (21.4)	1315 (18.3)

Among the 4591 children under 5 years old, 1167 (25.4%) were diagnosed clinically, without documentation of laboratory confirmation in the register. However, there were notable outliers; for example, Kankan, which had 403 cases (86.9%) registered with no laboratory confirmation, and Nzérékoré, which had 21 cases (3.4%) with no laboratory confirmation. Among this group, 51.5% of malaria diagnoses were confirmed by RDT and 23.0% by microscopy. Among the 7195 patients 5 years or older, 27.3% received a diagnosis of uncomplicated malaria without documentation of laboratory confirmation noted in the register. The rate of clinical diagnosis ranged from 4.6% in Nzérékoré to 73.7% in Kankan. Among this group, 47.4% of cases were confirmed with RDT (Table 1).

Across the eight regions, 35.9% of malaria cases under 5 years of age and 25.1% of cases 5 years of age or older were recorded as severe malaria, defined as diagnosis of or treatment for severe malaria (parenteral therapy given) documented in the register. The regions with the highest rate of severe malaria diagnosis included Faranah, with 57.0% of patients under 5 and 33.2% of patients 5 years or older diagnosed with severe malaria, and Labé, with 51.0% of patients under 5 and 34.4% of patients 5 and older diagnosed with severe malaria. The region with the lowest rate of severe malaria was Conakry, with a diagnosis of severe malaria for 6.6% of patients under 5 and 7.7% of patients 5 years of age and older. The diagnosis of uncomplicated malaria represented 64.1% of malaria cases among children under 5 and 74.9% of those 5 years of age and older (Table 1).

Among all ages and regions, ACT was prescribed for the majority of cases of uncomplicated malaria, confirmed or not (83.5%) (Table 2). Among patients documented as having uncomplicated malaria, 79.3% were prescribed a non-commercial ACT: 49.6% with ASAQ, 27.7% with AL, and 2.0% were not specified. Commercial ACT was used to treat 4.2% of patients diagnosed with uncomplicated malaria. It was also found that 3.8% of people prescribed ACT had a negative malaria diagnostic test, against the recommendation outlined in the national protocol for malaria treatment. Among children under 5 years, the proportion of treatment of malaria-negative cases was 2.8%; for those over 5 years, it was 4.2%. Additionally, there was no information about treatment prescribed for 15.9% of patients diagnosed with malaria in the reviewed registers (Table 2).

**Table 2:** Frequency of ACT prescription for uncomplicated malaria, by region, evaluated by a retrospective review of health facility registers from 1 May to 31 July, 2016, Guinea

	Boké	Kindia	Faranah	Labé	Nzérékoré	Mamou	Conakry	Kankan	Total
	n (%)	n (%)	n (%)	n (%)	n (%)	n (%)	n (%)	n (%)	n (%)
**Treatment for uncomplicated malaria**	**n = 751**	**n = 1223**	**n = 970**	**n = 742**	**n = 1254**	**n = 1797**	**n = 1393**	**n = 694**	**n = 8824**
ASAQ	594 (79.1)	1113 (91.0)	170 (17.5)	370 (49.9)	458 (36.5)	1302 (72.5)	334 (24.0)	40 (5.8)	4381 (49.6)
AL	74 (9.9)	0	379 (39.1)	102 (13.7)	273 (21.8)	302 (16.8)	970 (69.6)	341 (49.1)	2441 (27.7)
ACT not specified	30 (4.0)	0	38 (3.9)	1 (0.1)	0	0	0	109 (15.7)	178 (2.0)
Commercial ACT	5 (0.7)	0	155 (16.0)	201 (27.1)	0	0	0	13 (1.9)	374 (4.2)
Non-ACT	48 (6.4)	0	1 (0.1)	0	1 (0.1)	0	0	1 (0.1)	51 (0.6)
Not documented	0	110 (9.0)	227 (23.4)	68 (9.2)	522 (41.6)	193 (10.7)	89 (6.4)	190 (27.4)	1399 (15.9)
**Confirmed uncomplicated malaria**	**n = 693**	**n = 1080**	**n = 591**	**n = 426**	**n = 1206**	**n = 1211**	**n = 1292**	**n = 141**	**n = 6640**
Treated with ACT	646 (93.2)	1054 (97.6)	515 (87.1)	387 (90.8)	703 (58.3)	1086 (89.7)	1207 (93.4)	102 (72.3)	5700 (85.8)
**Suspected uncomplicated malaria, negative test**									
Treated with ACT	14	5	26	125	5	7	44	52	278
**Suspected uncomplicated malaria, without reported test (clinically diagnosed)**	**n = 44**	**n = 131**	**n = 324**	**n = 185**	**n = 39**	**n = 577**	**n = 53**	**n = 487**	**n = 1840**
Treated with ACT	43 (97.8)	54 (41.2)	201 (62.0)	162 (87.6)	23 (59.0)	511 (88.6)	53 (100)	349 (71.7)	1396 (75.9)

### Interviews and follow up of patients prescribed ACT

During the study period, 2019 individuals were diagnosed with uncomplicated malaria and were included on day 1 of the study. Among these patients, 1830 (90.6%) were present on the last day of follow up (Table 4). Among patients diagnosed with uncomplicated malaria, 2.1% reported receiving messages regarding the importance of repeating the initial dose if vomiting occurs, and 9.1% reported hearing about signs that necessitate an immediate return to the health facility. The messages most frequently received by patients diagnosed with uncomplicated malaria were about the ACT regimen (94.6%), taking the ACT with a sugary drink and during a meal (65.7%), and methods of protection against malaria (56.9%) (Table 3).

**Table 3:** Proportion of key messages received by patients with uncomplicated malaria by region, 2016, Guinea

	Boké	Kindia	Faranah	Labé	Nzérékoré	Mamou	Conakry	Kankan	Total
	n (%)	n (%)	n (%)	n (%)	n (%)	n (%)	n (%)	n (%)	n (%)
	n = 211	n = 191	n = 252	n = 209	n = 237	n = 254	n = 231	n = 245	N = 1830
*Messages regarding case management*									
Direct observation of first dose at health facility	211 (100)	76 (39.8)	251 (99.6)	13 (6.2)	0	10 (3.9)	9 (3.9)	76 (39.8)	798 (43.6)
ACT dosage	211 (100)	191 (100)	252 (100)	203 (97.1)	145 (61.2)	254 (100)	231 (100)	245 (100)	1732 (94.6)
Repeat the initial dose if vomiting occurs	0	3 (1.6)	15 (6.0)	4 (1.9)	1 (0.4)	4 (1.6)	2 (0.9)	10 (4.1)	39 (2.1)
Information on possible side effects of ACT	5 (2.4)	45 (23.6)	52 (20.6)	80 (38.3)	1 (0.4)	104 (40.9)	21 (9.1)	4 (1.6)	312 (17.0)
Signs that necessitate an immediate return to health facility	19 (9.0)	0	56 (22.2)	1 (0.5)	7 (3.0)	80 (31.5)	3 (1.3)	1 (0.4)	167 (9.1)
Importance of follow-up visit	25 (11.8)	4 (2.1)	154 (61.1)	0	1 (0.4)	4 (1.6)	3 (1.3)	197 (80.4)	388 (21.2)
Take the ACT with a sugary drink during a meal	147 (69.7)	185 (96.9)	82 (32.5)	176 (84.2)	105 (44.3)	130 (51.2)	140 (60.6)	237 (96.7)	1202 (65.7)
First dose supervised by healthcare provider	0	0	3 (1.2)	17 (8.1)	4 (1.7)	254 (100)	0	7 (2.9)	285 (15.6)
Explanation of how to protect against malaria	200 (94.8)	72 (37.7)	139 (55.2)	72 (34.4)	14 (5.9)	249 (98.4)	50 (21.6)	245 (100)	1041 (56.9)
*Prevention messages*									
Use of long lasting insecticidal mosquito net	208 (98.6)	78 (40.8)	143 (56.7)	44 (21.1)	4 (21.1)	244 (96.1)	57 (24.7)	238 (97.1)	1016 (55.5)
Environmental hygiene and sanitation	1 (0.5)	7 (3.7)	27 (10.7)	11 (5.3)	11 (5.3)	25 (9.9)	1 (0.4)	109 (44.5)	183 (10.0)

Among the patients prescribed either ASAQ or AL (n = 1830), 23.4% reported experiencing at least one adverse event. The proportion of adverse events was significantly higher (p < 0.001) for those prescribed with ASAQ (34.1%) compared with those prescribed with AL (8.8%). Only the proportion of those reporting gastro-intestinal adverse events was not statistically significantly different between the two types of ACT. Vomiting, fatigue, dizziness, and drowsiness were all reported more frequently for those prescribed ASAQ in comparison with AL (Table 4).

**Table 4:** Comparison of proportions of patient adherent to treatment and reported experiencing side effects by type of ACT prescribed, 2016, Guinea

	ASAQ	AL	p*
	(n = 1060)	(n = 770)	
	n (%)	n (%)	
Proportion of adherent patients	1003 (94.6)	742 (96.4)	0.08
Reported side effects	361 (34.1)	68 (8.8)	< 0.001
Vomiting	57 (5.4)	22 (2.9)	0.009
Fatigue	287 (27.1)	14 (1.8)	< 0.001
Dizziness	42 (4.0)	10 (1.3)	0.001
Gastro-intestinal trouble	47 (4.4)	23 (3.0)	0.11
Drowsiness	50 (4.7)	6 (0.8)	< 0.001
Other side effect^a^	14 (1.3)	5 (0.6)	< 0.16

* χ^2^ test

^a^Anorexia (n = 4), ringing in the ears (n = 3), facial puffiness (n = 2), insomnia (n = 1), cough (n = 1), itch (n = 1), headache (n = 2), fever (n = 1), chills (n = 4)

Overall, 95.4% of patients diagnosed with uncomplicated malaria reported completing treatment without interruption, 94.6% for patients prescribed ASAQ and 96.4% for those prescribed AL. Among patients prescribed ACT, 85 (4.6%) were found to be non-adherent. In the final multiple logistic regression model, region, age, and experience of adverse events were the variables found to be statistically significantly associated with non-adherence (Additional file 1: Table S2).

According to the multivariate logistic regression model, the odds of non-adherence for patients older than 5 were almost twice the odds of those 5 years of age or under (adjusted OR: 1.7, 95% CI 1.1, 2.9), holding region and experience of adverse events constant. The odds of non-adherence among those who experienced adverse events was almost three times the odds for those who did not (adjusted OR: 2.9, 95% CI 1.8–4.6), holding age and region constant (Table 5).

**Table 5:** Univariate and multivariate analyses of factors associated with non-adherence to ACT, 2016, Guinea

Variable	Non-adherent	Unadjusted OR (95% CI)	p-value	Adjusted OR (95% CI)	p-value
	n (%)				
*Region*			0.05		0.001
Kankan	1 (0.4)	Ref.		Ref.	
Boké	7 (3.3)	8.4 (1.0, 68.6)		4.5 (0.5, 38.0)	
Kindia	12 (6.3)	16.4 (2.1, 126.9)		9.9 (1.2, 78.6)	
Faranah	21 (8.3)	22.2 (3.0, 166.2)		14.9 (1.9, 113.5)	
Labé	19 (9.1)	24.4 (3.2, 183.9)		16.2 (2.1, 123.4)	
Nzérékoré	8 (3.4)	8.5 (1.1, 68.7)		7.5 (0.9, 60.9)	
Mamou	5 (2.0)	4.9 (0.6, 42.2)		3.0 (0.3, 26.3)	
Conakry	12 (5.2)	13.4 (1.7, 103.7)		7.8 (0.9, 61.8)	
*Patient age (years)*			0.04		< 0.001
≤ 5	25 (3.4)	Ref.		Ref.	
> 5	60 (5.5)	1.6 (1.0, 2.6)		1.7 (1.8, 4.6)	
*Side effects*			< 0.001		< 0.001
No	43 (3.1)	Ref.		Ref.	
Yes	42 (9.8)	3.4 (2.2, 5.3)		2.9 (1.8, 4.6)	
*Patient gender*			0.55		
Female	43 (4.4)	Ref.			
Male	42 (5.0)	1.1 (0.7, 1.8)			
*Facility type*					
Hospital	34 (5.0)				
Urban health centre	36 (5.2)				
Rural health centre	15 (3.3)				
*Education level*			0.06		
Some education	40 (5.9)	Ref.			
No education	45 (3.9)	0.7 (0.4, 1.0)			
*Marital status*			0.31		
Married	23 (5.6)	Ref.			
Single	62 (4.4)	0.8 (0.5, 1.3)			
*ACT prescribed*			0.05		
AL	28 (3.6)	Ref.			
ASAQ	57 (5.4)	1.5 (0.9, 2.4)			

### Interviews of prescribers

In the 24 health facilities, 115 prescribers were interviewed. Among the prescribers, 65.2% were interviewed at hospitals and 34.8% at health centres. The mean age of prescribers was 38.9 years (sd: 11.7) (Additional file 1: Table S3). The majority (95.7%) of prescribers reported having knowledge of the national policy for malaria treatment. Additionally, 87.0% reported having received a supervision visit in the previous 6 months, and 45.2% reported receiving malaria-related training in the previous 6 months. A malaria-related training document was available for 66.1% of prescribers, and 54.8% prescribers had a treatment algorithm available.

Among prescribers interviewed, 25.2% reported having a very favourable opinion of the national malaria treatment policy, and 63.5% reported having a favourable opinion. Five prescribers (4.3%) reported having little favourability towards the ACT policy. The reason for this was adverse events associated with ASAQ for four, and one prescriber noted: “(ACT) gives good results but these medications are imposed on us” (Additional file 1: Table S4).

In the 24 health facilities included in the study, 43 drug dispensers were interviewed. The majority were women (65.1%); the mean age was 40.6 years (sd: 9.9) and most worked at a point of sale (58.1%). Drug dispensers reported having training as a technical health agent (51.2%), pharmacist (23.3%), nurse (18.6%), and other (7.5%). Among the interviewed drug dispensers, 95.3% reported having a malaria-related supervision in the past 6 months and 86.0% reported having knowledge of the national malaria treatment protocol. Training documents on malaria treatment were available for 42.9% of drug dispensers. This proportion was higher in health centres (59.1%) than in hospitals (25.0%).

Hesitation from patients about the taking an ACT was observed by 32.6% of drug dispensers interviewed, and of the drug dispensers that observed hesitation, 100% reported the hesitation as being about ASAQ (as opposed to AL or other). This was reported more often in hospitals (45%) then in health centres (21.7%). Additionally, 25.6% of drug dispensers reported being reluctant themselves to dispense ASAQ to patients due to side effects (Additional file 1: Table S5).

## Discussion

In this study, it was found that most patients diagnosed with uncomplicated malaria were prescribed an ACT, which is in line with the national malaria treatment policy. Factors associated with non-adherence included region, age and experience of adverse events. Additionally, most prescribers and drug dispensers reported favourability towards the national treatment guidelines, but some had reservations about the prescription of ACT. Although there were more reported adverse events associated with ASAQ when compared with AL, both prescribers and patients were found to be mostly adherent to ACT for the treatment of malaria, regardless of ACT type.

This study found that the frequency of ACT prescription for uncomplicated malaria (confirmed or not) was 83.7%. This percentage reached 85.8% for patients that had a laboratory confirmation documented in the register. Additionally, it was found that very few patients were treated with a non-ACT (monotherapy). These encouraging results call into question the findings of the Multi Indicator Cluster Survey done in 2016 that found that only 16.5% of anti-malarial treatments given to children under 5 were ACT [4].

Even though the prescription of ACT among confirmed patients was high, it was found that 278 patients with a negative RDT were also prescribed an ACT, and this proportion was higher among patients over 5 years old. This difference of practices by age could be due to the higher proportion of adults treated at hospitals, which had overall higher proportions of patients with a negative diagnostic test being treated. Additionally, 20.8% of patients were clinically diagnosed with uncomplicated malaria without a reported test. This departure from the national malaria treatment guidelines, despite the majority of providers interviewed indicating knowledge of them, indicates that more should be done to ensure that there are no stock outs of RDTs and that healthcare providers are properly trained and supervised.

A high proportion of diagnosed severe malaria was found documented in the registers. In children under 5 years old, 35.9% of malaria cases were documented as severe. In two out of the eight regions (Faranah and Labé), most of malaria cases in under 5 s were documented as severe. It is unclear from this finding if these diagnoses were indication of ‘true’ severe malaria or if there is a high rate of misdiagnosed severe malaria. There are risks associated with severe malaria treatment, and unnecessary use of these treatments should be avoided. Further investigation of this finding is critical to prevent severe morbidity and mortality and to confirm that practitioners are adhering to diagnostic guidelines.

Patient interviews in this survey indicate that patients are not receiving and/or retaining the information needed with a prescription of ACT. Fewer than one in five patients recalled receiving information about possible adverse events by the prescriber, and fewer than one in ten recalled receiving information about signs that would necessitate a return to the health facility. Only about half of patients recalled receiving information about how to prevent malaria. Without complete and accurate information, patients could be less likely to adhere to the entire treatment regimen and/or be at risk for repeated malaria infections. To ameliorate the lack of complete information provided to patients, training of prescribers could include role-playing and mock patients to practice patient counselling, perhaps in addition to a job aid. Additionally, malaria-related supervision could include observations of consultations along with a checklist of all messages that should be received by a patient.

With the extensive use of ACT in the endemic areas, including Guinea, surveillance of safety of anti-malarial medication is critical. Almost one out of four patients in this study (23.4%) reported experiencing adverse events. Patients diagnosed with uncomplicated malaria who reported adverse events had almost three times the odds of interrupting treatment than those who did not (holding age and region constant). The most common side effects of fatigue, drowsiness and nausea/vomiting associated with ASAQ were consistent with studies evaluating the safety of ASAQ [16–18]. Even if the adverse events were minor, it is important that prescribers consider potential adverse events when educating patients upon prescribing ACT. Despite the elevated levels of adverse events associated with ASAQ, adherence was not found to be significantly different between ASAQ and AL.

While some studies have compared AL with other ACT or malaria medication, this was the first published quantitative study to compare adherence between ASAQ and AL [19]. A qualitative study completed in Ghana in 2015 found that participants preferred AL to ASAQ and dihydroartemisinin piperaquine (DP). Participants who reported being prescribed ASAQ described adverse events related to the drug impacting their ability to adhere to the full regimen [12]. Other studies examining adherence to ACT in sub-Saharan Africa found that adherence was associated with education, age and taking the first dose under supervision of a healthcare worker [19–23]. In a study conducted in Senegal, 227 symptoms were reported among 123 patients on ACT. Most of these symptoms (46.7%) were related to digestive and abdominal problems. The present study reinforces the conclusions that others found [24, 25] that provide evidence to advocate for stronger pharmacovigilance related to adverse events or adverse events associated with ACT. Currently, there is no formal surveillance system for drug-related adverse events in Guinea. Incorporating adverse event reporting into the routine reporting system through the District Health Information System-2 (DHIS-2), which is in the process of being implemented in Guinea, could strengthen pharmacovigilance surrounding ACT and other drugs.

Most prescribers and drug dispensers had positive opinions regarding the national guidelines for treatment, but some did report having reservations about using ACT to treat uncomplicated malaria because of perceived side effects. While ASAQ is no longer the only first-line ACT used to treat uncomplicated malaria in Guinea, evidence from this study suggests that AL does have a preferable side effect profile, but given very high overall adherence, there is no evidence of a difference in adherence between the two drugs.

Patients 5 years or older had two times the odds of those younger than 5 of being non-adherent (AOR 1.7, holding region and adverse events constant), indicating that caregivers and parents might have a perception of children under 5 being at higher risk of severe disease than those older, or perhaps those older than 5 are more likely to refuse to take ACT.

The accuracy of the information extracted for the retrospective registry review to determine prescription practices cannot be guaranteed. The drivers behind the described prescription practices cannot be determined using this method. The availability of commodities including RDTs and ACT can be a determinant of diagnostic and prescription practices; without stock data from the relevant health facilities during the study period, there was no way to explore this relationship. Adherence in this study was based on patient self-report, and about 10% of patient interviews were conducted by telephone, limiting observation of the blister pack of ACT. Finally, the survey procedure of asking patients to save the blister pack at the end of their treatment could introduce the systematic bias to the study, potentially inadvertently changing patient behaviour by implying that adherence would be checked.

## Conclusion

This study provides insight into prescriber practices regarding the diagnosis and treatment of malaria as well as adverse events and patient-adherence to two types of ACT in Guinea. Other countries considering changing treatment policies or first-line ACT might consider carrying out similar studies to measure potential impact on prescriber attitudes and patient behaviour regarding a change of policy or ACT. Future studies could be improved by adding data about the stock of malaria diagnostic and treatment commodities. It could also be simplified by reducing the scope of the retrospective registry review.

## Additional files

**Additional file 1.** Supplemental tables describing descriptions of interviewees and practices related to ACT adherence.

**Additional file 2.** Questionnaires and template for recording information from registers used for data collection.

## Abbreviations

ACT: artemisinin-based combination therapy
AL: artemether/lumefantrine
ASAQ: artesunate/amodiaquine
DHIS2: District Health Information System 2
DP: dihydroartemisinin piperaquine
NMCP: National Malaria Control Programme
RDT: rapid diagnostic test
SMC: seasonal malaria chemoprevention
SP: sulfadoxine–pyrimethamine
WHO: World Health Organization
