# Supplementary material for: Prescriber practices and patient adherence to artemisinin-based combination therapy for the treatment of uncomplicated malaria in Guinea, 2016
Source: Malar J. 2019 Jan 25;18:23. doi: 10.1186/s12936-019-2664-7 (PMC6347834; doi:10.1186/s12936-019-2664-7)
Supplement: Supplementary file 1 — Additional file 1. Supplemental tables describing descriptions of interviewees and practices related to ACT adherence. [file 12936_2019_2664_MOESM1_ESM.docx]

**Additional files**

**Additional file Table S1: Description of sampled health facilities, 2016, Guinea**

| **Region** | **Randomly selected district** | **Sampled health facilities** |
| --- | --- | --- |
| Boké | Boké | RH Boké, UHS Koulifanya, RHS Sangaredi |
| Kindia | Coyah | PH Coyah, UHS Fily, RHS Maneah |
| Labé | Labé | RH Labé, UHS Leysarè, RHC Dionfo |
| Mamou | Mamou | RH Mamou, UHS Séré, RHC de Saramoussaya |
| Faranah | Kissidougou | PH Kissidougou, UHS Dares salam, RHS Gbangbandou |
| Kankan | Siguiri | PH Siguiri, UHS Siguirikoro 1, RHC Niandakoro |
| N’zérekoré | Macenta | RH Macenta, UHS Hermakono, RHC Balizia |
| Conakry | Ratoma | CMC Ratoma, UHS Sonfonia, UHS Wanidara |
| RH: Regional hospital, PH: Prefectural Hospital, UHS: Urban health center, RHC: Regional health center, CHC: Communal Medical Center | | |

**Additional file Table S2: Description of practices and side effects experienced by patients diagnosed with uncomplicated malaria and prescribed ACT by region, 2016, Guinea**

|  | Boké | Kindia | Faranah | Labé | Nzérékoré | Mamou | Conakry | Kankan | Total |
| --- | --- | --- | --- | --- | --- | --- | --- | --- | --- |
|  | n(%) | n(%) | n(%) | n(%) | n(%) | n(%) | n(%) | n(%) | n(%) |
|  | **n=211** | **n=191** | **n=252** | **n=209** | **n=237** | **n=254** | **n=231** | **n=245** | **N=1830** |
| **Adherence and response to treatment** |  |  |  |  |  |  |  |  |  |
| Started treatment | 211 (100) | 191 (100) | 252 (100) | 209 (100) | 237 (100) | 254 (100) | 231 (100) | 245 (100) | 1830 (100) |
| Completed treatment without interruption | 204 (96.7) | 179 (93.7) | 231 (91.7) | 190 (90.9) | 229 (96.6) | 249 (98.0) | 219 (94.8) | 244 (99.6) | 1745 (95.4) |
| Reported experiencing side effects | 90 (42.7) | 71 (37.2) | 80 (31.7) | 54 (25.8) | 8 (3.4) | 66 (26.0) | 60 (26.0) | 0 | 429 (23.4) |
| **Distribution of side effects** |  |  |  |  |  |  |  |  |  |
| Vomiting | 6 (2.8) | 5 (2.6) | 29 (11.5) | 10 (4.8) | 1 (0.4) | 15 (5.9) | 13 (5.6) | 0 | 79 (4.3) |
| Fatigue | 86 (40.8) | 67 (35.1) | 51 (20.2) | 38 (18.2) | 3 (1.3) | 28 (11.0) | 28 (12.1) | 0 | 301 (16.4) |
| Dizziness | 3 (1.4) | 12 (6.3) | 0 | 3 (1.4) | 1 (0.4) | 19 (7.5) | 14 (6.1) | 0 | 52 (2.8) |
| Gastro-intestinal trouble | 0 | 1 (0.5) | 20 (7.9) | 7 (3.3) | 0 | 26 (10.2) | 16 (6.9) | 0 | 70 (3.8) |
| Drowsiness | 7 (3.3) | 14 (7.3) | 18 (7.1) | 0 | 0 | 9 (3.5) | 8 (3.5) | 0 | 56 (3.1) |
| Other side effect* | 0 | 0 | 2 (0.8) | 5 (2.4) | 3 (1.3) | 2 (0.8) | 7(3.0) | 0 | 19 (1.0) |
| **Steps taken upon experiencing side effects** | **n = 90** | **n=71** | **n= 80** | **n=54** | **n=8** | **n=66** | **n=60** | **n=0** | **N=429** |
| Stopped treatment | 5 (5.6) | 5 (7.0) | 2 (2.5) | 4 (7.4) | 1 (12.5) | 0 | 1 (1.7) | 0 | 18 (4.2) |
| Consulted treating clinician | 0 | 8 (11.3) | 24 (30.0) | 1 (1.9) | 7 (87.5) | 27 (40.9) | 14 (23.3) | 0 | 81 (18.9) |
| Consulted other clinician | 0 | 4 (5.6) | 4 (5) | 3 (5.6) | 0 | 0 | 0 | 0 | 11 (2.6) |
| Continued treatment | 85 (94.4) | 54 (76.1) | 50 (62.5) | 46 (85.2) | 0 | 39 (59.1) | 45 (75.0) | 0 | 319 (74.4) |
| * anorexia (n=4), ringing in the ears (n=3), facial puffiness (n=2), insomnia (n=1), cough (n=1), itch (n=1), headache (n=2), Fever (n=1), chill (n=4) | | | | | | | | | |

**Additional file Table S3: Description of prescribers interviewed, Guinea, 2016**

|  | Hospital | Health Center | Total |
| --- | --- | --- | --- |
|  | n (%) | n (%) | n (%) |
| Age Group |  |  |  |
| <40 years | 48 (64.0) | 22 (55.0) | 70 (60.9) |
| ≥40 years | 27 (36.0) | 18 (45.0) | 45 (39.1) |
| Sex |  |  |  |
| Male | 58 (77.3) | 20 (50.0) | 78 (67.8) |
| Female | 17 (22.7) | 20 (50.0) | 37 (32.2) |
| Prescriber type |  |  |  |
| Doctor | 53 (70.7) | 8 (20.0) | 61 (53.0) |
| Nurse | 8 (10.7) | 9 (22.5) | 17 (14.8) |
| Health Technician | 5 (6.7) | 20 (50.0) | 25 (21.7) |
| Midwife | 5 (6.7) | 0 | 5 (4.3) |
| Other | 4 (5.3) | 3 (7.5) | 7 (6.1) |

**Additional file Table S4: Distribution of prescribers by opinion regarding the application of the national malaria control policies by health facility type, Guinea, 2016**

|  | Hospital | Health Center | Total | p-value |
| --- | --- | --- | --- | --- |
|  | n (%) | n (%) | n (%) |  |
|  | n=75 | n=40 | N=115 |  |
| Knowledge of the national malaria treatment protocol | 73 (97.3) | 37 (92.5) | 110 (95.7) | 0.23 |
| Trained on malaria in the previous 6 months | 33 (44.0) | 19 (47.5) | 52 (45.2) | 0.72 |
| Documents on malaria treatment available | 53 (70.7) | 23 (57.5) | 76 (66.1) | 0.15 |
| Received malaria-related supervision in the previous 6 months | 60 (80.0) | 40 (100) | 100 (87.0) | 0.002 |
| Algorithm on malaria treatment available | 36 (48.0) | 27 (67.5) | 63 (54.8) | 0.45 |

**Additional file Table S5: Prescriber & dispenser opinions on the national malaria treatment policy (N=115)**

|  | Hospital | Health Center | Total |
| --- | --- | --- | --- |
|  | n (%) | n (%) | n (%) |
| **Prescribers** |  |  |  |
| Yes, totally in agreement with the use of ACT | 67 (89.3) | 37 (92.5) | 104 (90.4) |
| Yes, partially in agreement with the use of ACT | 8 (10.7) | 3 (7.5) | 11 (9.6) |
|  |  |  |  |
| Very favorable opinion of malaria treatment protocol | 19 (25.3) | 10 (25.0) | 29 (25.2) |
| Favorable opinion of malaria treatment protocol | 50 (66.7) | 23 (57.5) | 73 (63.5) |
| Little favorability towards malaria treatment protocol | 3 (4.0) | 2 (5.0) | 5 (4.3) |
| Unfavorable opinion of malaria treatment protocol | 0 | 0 | 0 |
| No opinion on malaria treatment protocol | 3 (4.0) | 5 (12.5) | 8 (7.0) |
| **Dispensers** |  |  |  |
| Often observes hesitation | 9 (45.0) | 5 (21.7) | 14 (32.6) |
| Rarely observes hesitation | 1 (5.0) | 7 (30.4) | 8 (18.6) |
| Does not observe hesitation | 10 (50.0) | 11 (47.8) | 21 (48.8) |
|  |  |  |  |
| Has training materials on malaria treatment | 2 (10.0) | 7 (30.4) | 9 (20.9) |
| Knowledge of the national protocol for malaria treatment | 16 (80.0) | 21 (91.3) | 37 (86.0) |
| Has had a malaria-related supervision in the past 6 months | 19 (95.0) | 22 (95.7) | 41 (95.3) |
