# Supplementary material for: Prescriber practices and patient adherence to artemisinin-based combination therapy for the treatment of uncomplicated malaria in Guinea, 2016
Source: Malar J. 2019 Jan 25;18:23. doi: 10.1186/s12936-019-2664-7 (PMC6347834; doi:10.1186/s12936-019-2664-7)
Supplement: Supplementary file 2 — Additional file 2. Questionnaires and template for recording information from registers used for data collection. [file 12936_2019_2664_MOESM2_ESM.docx]

**QUESTIONNAIRE USAGER**

*Encerclez les bonnes réponses*

| **Informations sur les structures sanitaires** | |
| --- | --- |
| PIS1. Numéro de fiche : | /___/___//___/Automatique |
| PIS2. Numéro de l’usager | /_____/ /_____/ /______/ /_______ /  Région district structure Numéro |
| PIS3. Nom du district sanitaire | ___ ___ ___ ___ ______ ___ ___ |
| PIS4. Nom du service sanitaire | Urgences médicales……………..………..…..1  Pédiatrie………………………..….……………2  Médecine générale……………..……….……..3  Maternité…………………………...…………...4  Consultation primaire curative…..……………5 |
| PIS5. Nom de la localité | ___ ___ ___ ___ ______ ___ ___ |
| PIS6. Type d’établissement sanitaire | Hôpital régional …………………………………….…….1  Hôpital préfectoral .……………………………….…….2  Centre Médical Communal ………………….………3  Centre de santé urbain..……………………….……..4  Centre de santé rural……………………………..……..5 |
| Numéro téléphone personnel | ___ ___ ___ ___ ______ ___ ___ |
| Numéro téléphone d’un proche | ___ ___ ___ ___ ______ ___ ___ |
| Adresse | ___ ___ ___ ___ ______ ___ ___ |
| PIS7. Date de Consultation | /___/___/______/ |
| PIS8. Date de debut de prise de CTA | /___/___/______/ |
| PIS9. Date de FIN de prise de CTA | /___/___/______/ |
| PIS10. Date d’interview | /___/___/______/ |

| **Information sur l’usager des CTA** | | | |
| --- | --- | --- | --- |
| PIU1.Quel est l’âge de l’usager ? | | Âge (en années révolues)  Age (en mois pour 0 à 11mois)……………………… |  |
| PIU2. Quel est le genre de L’usager ? | | Masculin………………………………………………………………1  Féminin………………………………………..……………………..2 |  |
| PIU3. Niveau d’instruction de l’usager? | | Non scolarisé……………………………………………………….1  Primaire ……………………………………………….…………….2  Secondaire ...………………………………..…………………….3  Supérieur………………………………………………………..…..4  Professionnel………………………………………………….…..5  Autre…………………………………………………………………...6 | Si réponse 6, précisez |
| PIU4. Profession de l’usager ? | | Employé……………………………………………………………..1  Sans emploi………………………………………………………..2  Libéral………………………………………….……………………..3  Autre……………………………………………………..…………..4 | Précisez pour toutes les options |
| PIU5. Situation matrimoniale de l’usager ? | | Célibataire ……………………………………….………..……….1  Marié …………………………………..…………………….……….2  Autre……………………………………………………………………3 | Si réponse 3, précisez |
| **Connaissance sur le paludisme simple** | | | |
| CP1. Avant votre venue dans la structure sanitaire, Avez-vous connaissance de l’existence de medicaments contre le paludisme ? | | Oui…………………………………………………….………….….…1  Non……………………………………………………………………..2 |  |
| CP2. si oui, pouvez-vous nous citer des exemples de médicaments contre le paludisme ? | | …………………………………………………………………………  ………………………………………………………………………… |  |
| CP3. Avant votre venue dans la structure sanitaire, Quels sont les principaux moyens pour éviter d’attraper le paludisme que vous connaissiez? | | Dormir sous moustiquaire ordinaire …. …………..……1  Dormir sous moustiquaire imprégnée…………….2  Utiliser les insecticides/ spirales …………………..3  Garder les alentours de la maison propre ………..4  Couper les herbes ……………………………........5  Eliminer les eaux stagnantes ………………………6  Pulvérisation des murs intérieurs ………………….7  Ne sait pas…………………………..……………….8  Autre (*préciser*)……………………………………….9 |  |
| **Diagnostic et traitement du Paludisme simple (PS)** | | | |
| DTP1. Motif de consultation ? | | Fièvre…………………………………………………………….…...1  Céphalée………………………………………………….…….…..2  Vomissement…………………………………………………..…3  Nausée………………………………………………………..……..4  Vertige………………………………………………………………..5  Courbature générale…………………………………………..6  Diarrhée……………………………………………………….……..7  Autre……………………………………………………………….….8 | Si réponse 8, précisez |
| - DTP2. Quel est le diagnostic de présomption ? | | Paludisme simple…………………………………………….…..1  Autre……………….…………………………………………………..2 | Si réponse 2, précisez |
| - DTP3. Quel est le diagnostic de certitude ? | | Paludisme simple…………………………………………….…..1  Autre……………………………………………………………………2 | Si réponse 2, précisez |
| DTP4. Le diagnostic du Paludisme simple a été effectué avec quel test ? | | TDR……………………………………………………………………..1  GE……………………………………………………………………….2  Autre…………………………………………………………………..3 | Vérifier dans le carnet du malade, si possible |
| - DTP5. Quel était le résultat du test ? | | Positif………………………………………………………..……….1  Négatif………………………………………………………..……..2  Non disponible……………………………………………..……3 |  |
| - DTP6. Quel a été le traitement prescrit ? | | ASAQ……………………………………………………………..……1  AL……………………………………………………………………..…2 | Précisez la posologie journalière |
| - DTP7. Le médecin vous a-t-il expliqué comment prendre le CTA ? | | Oui…………………………………………………….………….….…1  Non……………………………………………………………………..2 |  |
| - DTP8. Le prescripteur vous a-t-il expliqué comment se protéger du paludisme ? | | Oui…………………………………………………….………….….…1  Non……………………………………………………………………..2 |  |
| - DTP9. Le prescripteur a-t-il pris les mesures suivantes lors de la dispensation? | | Première dose supervisée par le prestataire……...1  Répéter la dose initiale si vomissement……………2  Informer sur les effets indésirables possibles des CTA….…………………………………………………3  Organiser une visite de suivi 2 jours après le traitement …………………………………..….……..4  Aucune mesure……………………………..………..5  Autres………………………………………..….……..6 |  |
| - DTP10. Le prescripteur vous a-t-il donne des conseils sur la prise des CTA? | | Prise de CTA avec boissons sucrées et au cours des repas………………………………………...……1  L’utilisation de la MILDA………………………………………2  L’observance du traitement médicamenteux…….3  Les signes nécessitant le retour immédiat …….…..4  L’importance de la visite de suivi………………………...5  L’hygiène du milieu de vie et l’assainissement…...6  Aucun conseil………………………………………………………7  Autres………………………………………………………………….8 |  |
| - DTP11. Le patient a-t-il pris ces comprimés (CTA) ? | | Oui…………………………………………………….………….….…1  Non……………………………………………………..……………..2 |  |
| - DTP12. Si oui, quelle était la posologie journalière ? | | Un comprimé……………………………………………….……..1  Deux Comprimés…………………………………….…………..2  Trois comprimés et plus………………………………………3 |  |
| - DTP13. La prise des CTA a-t-il connu des interruptions journalières | | Oui…………………………………………………….………….…….1  Non……………………………………………………………………..2 | 1⇨ Précisez le nombre de jours |
| - DTP14. Si Oui, pourquoi le patient n’a-t-il pas pris ces comprimés (CTA) ? | | La non-croyance à l’efficacité des CTA………………...1  Rupture des CTA………………………………………………….2  Sur le conseil d’un proche……………………………………3  Autres………………………………………………………………….4 | Si réponse 4, précisez |
| - DTP15. Le patient a-t-il conservé sa tablette ? | | Oui…………………………………………………….………….……1  Non…………………………………………………………………….2 | 1⇨question  suivante  2⇨ dtp18 |
| - DTP16. Si oui, y a-t-il des restants de comprimés dans la tablette ? | | Oui…………………………………………………….………….……1  Non…………………………………………………………………….2 |  |
| - DTP17. Si oui, quelles sont les raisons des restants de comprimés dans la tablette ? | | Amélioration de l’état de santé…………………………..1  Effets indésirables……………………………………………….2  Posologie mal comprise……………………………………….3  Autre raison……………………………….…………….………….4 | Si réponse 4, précisez |
| - DTP18. Si Le patient n’a pas conservé sa tablette, a-t-il pris la totalité des comprimés ? | | Oui…………………………………………………….………….……1  Non…………………………………………………………………….2 |  |
| **Effets indésirables observés par l’usager** | | | |
| - EIU1. Le patient a-t-il présenté des effets indésirables ? | Oui……………………………………………………..….………………1  Non………………………………………………….…..……………….2 | |  |
| - EIU2. Si oui, quels étaient les effets indésirables observés par l’usager ? | Vomissement……………………………………………………..….1  Asthénie physique…………………………………………….…..2  Vertige…………………………………………………………………..3  Nausée……………………………………………………………….….4  Trouble gastro-intestinal………………………………….…...5  Autres…………………………………………………………….….….6 | | Si réponse 6, précisez |
| - EIU3. Si oui (eiu1), Quelle a été la conduite tenue face à ces effets indésirables ? | Arrêt du traitement…………………………………….…………1  Recours au prescripteur traitant……………………….……2  Recours à un autre prescripteur………………….…………3  Recours à la pharmacopée………………………….…………4  Autres………………………………………………………….………..5 | | Si réponse 5, précisez |
| - EIU4. Le patient a-t-il une allergie connue ? | Oui……………………………………..…………………………….……1  Non……………………………………..…………………………….….2 | | Si Oui, précisez |
| - EIU5. Le patient avait-t-il pris d’autre médicament avec les CTA ? | Oui……………………………………..…………………………………1  Non……………………………………..………………………….…….2 | |  |
| - EIU6. Si oui, citez ces médicaments ? | ________________________________________ | |  |
| **Avis de l’usager sur les CTA** | | | |
| AUC1. Quels est l’avis de l’usager sur les CTA ? | Bon………………………………………………………….…………….1  Acceptable…………………………………………………………….2  Mauvais…………………………………………………………………3  Pas d’avis………………………………………………………………4 | |  |
| AUC2. Si acceptable, pourquoi ? | ____ _____ _____________________________ | |  |
| AUC3. Si mauvais, pourquoi ? | ________________________________________ | |  |
| AUC4. Comment l’usager juge-t-il son état de santé ? | Guéri…………………………………………………………………….1  Amélioré……………………………………………………………...2  Pas d’amélioration……………………………………………….3  Décédé………………………………………………………………...4 | |  |
| AUC5. S’il n’y a pas eu d’amélioration, l’usager a-t-il eu recours à d’autre alternative ? | Oui…………………………………………………………………………1  Non……………………………………………………………………….2 | |  |
| AUC6. Si oui, précisez lesquels? | _____ ____ _____ ______ _____ ______ | |  |
| AUC7. En quelle médecine croyez-vous le plus ? | Médecine traditionnelle…………………………………..….1  Médecine moderne………………………………………………2  Pas d’avis………………………………………..……………………3 | |  |

MERCI

**QUESTIONNAIRE PRESCRIPTEUR**

*Encerclez les bonnes réponses*

| **Information sur les structures sanitaires** | |
| --- | --- |
| PIS1. Numéro de fiche | /___/___//___/Automatique |
| PIS2. Numéro du personnel | /_____/ /_____/ /______/ /_______ /  Région district structure Numéro |
| PIS3. Nom du district sanitaire | ___ ___ ___ ___ ______ ___ ___ |
| PIS4. Nom du service sanitaire | Urgences médicale…………………..……..…..1  Pédiatrie……………………………….…………2  Médecine générale…………………….………..3  Maternité………………………………….……...4  Consultation primaire curative…………………5 |
| PIS5. Nom de la localité | ___ ___ ___ ___ ______ ___ ___ |
| PIS6. Type d’établissement sanitaire | Hôpital régional..………………………….…..1  Hôpital préfectoral .……………………........….2  Centre Médical Communal………..….………..3  Centre de santé urbain.………………………..4  Centre de santé rural…………………….……..5 |
| PIS7. Date de l’interview | /_____/_____/________/ |

| **Information sur les prescripteurs** | | |
| --- | --- | --- |
| PIP1. Niveau de formation du prescripteur ? | Médecin……………………..……………………………………1  Infirmier.…………………….……………….……………………2  ATS ……………………………………………..………………3  Sage-femme…………………….…….……….…………………4  Autre…………………………….…….…………………………..5 | Si réponse 5, précisez |
| PIP2. Quel est le genre du prescripteur ? | Masculin………………………………………..…………………1  Féminin……………………………..……………………………..2 |  |
| PIP3. Quelle est votre situation matrimoniale ? | Marié………………………………………………………………1  Célibataire………………………………...………………………2  Autre……………………………………………………………….3 |  |
| PIP4. Quel est l’âge du prescripteur ? | Âge (en années révolues)………………………..……………………… |  |
| PIP5. Combien d’année d’expérience cumulez-vous ? | /_______/________/ |  |
| PIP6. Au cours des six (6) derniers mois, avez-vous suivi une formation sur le paludisme ? | Oui…………………………………..…………………………….1  Non………………………………………………………………..2 | 1⇨Q suivante  2⇨Q CAP1 |
| PIP7. Quels étaient les thèmes de formation? | ___ ___ ___ ___ ______ ___ ___ ___ ______ ___  ___ ___ ___ ___ ______ ___ ___ ___ ______ ___  ___ ___ ___ ___ ______ ___ ___ ___ ______ ____ |  |

| **Connaissance, Attitude et Pratique** | | |
| --- | --- | --- |
| CAP1. Avez-vous connaissance du protocole national de prise en charge actuel du paludisme ? | Vague……………………………………………………………..0  Oui………………...……………………………………………….1  Non……………….………………………………………………..2 |  |
| CAP2. Si Vague ou oui, Quelle est votre opinion de ce nouveau protocole de pec du paludisme ? | Peu favorable….……………………...………………………….1  Favorable …..………………….……...………………………….2  Très favorable………………………...………………………….3  Défavorable…………………………….…………………………4  Pas d’avis…………………………….……………………...……5 |  |
| CAP3. Quelles sont les raisons ? | ___ ___ ___ ___ ______ ___ ___ ___ _____ ___ |  |
| CAP4. Quelle est votre source d’information du nouveau protocole ? | ___ ___ ___ ___ ___ ___ ___ ___ ___ ______ ___ |  |
| CAP5. Disposez-vous des documents de formation sur la prise en charge du paludisme ? | Oui…………………………………………………………….…..1  Non………………………………………………………………..2 | 1⇨vérifiez et marquer vu. |
| CAP6. La structure a reçu une mission de supervision sur le paludisme du niveau supérieur durant les six derniers mois ? | Oui…………………………………………………………….…..1  Non………………………………………………………………..2 | 1⇨vérifiez et marquer vu. |
| CAP7. Votre structure dispose-telle des affiches d’algorithme de la prise en charge du paludisme ? | Oui………………………………..………………………………..1  Non……………………………..………………………………….2 | 1⇨vérifiez et marquer vu. |
| CAP8. Quelles sont les différents types de CTA que vous connaissez ? | AL…………………………….……………………………………1  ASAQ…………………….………………………………………..2  Artemisinin-SP……………………………………………………3  Dihydroartemisinin-piperaquine.....…………………………….4  Artesunate-Mefloquine.....………………...…………………….5  Autre.....…………………………………………………………...6 | Si réponse 6, précisez |
| CAP9. Quelles sont les différentes présentations de CTA que vous connaissez ? | Comprimés………………….……………………………………1  Sirop………………………….……………………………………2  Suppositoire………………….…….…………..…………………3  Poudre………………….…………………………………………4  Injectable……………….………...……………….………………5  Autre………………………….……………………………………6 | Si réponse 6, précisez |
| CAP10. Quelles sont les différents types de CTA qui sont disponibles dans votre établissement ? | AL…………………………….……………………………………1  ASAQ…………………….………………………………………..2  Artemisinin-SP……………………………………………………3  Dihydroartemisinin-piperaquine.....…………………………….4  Artesunate-Mefloquine.....………………...…………………….5  Autre.....…………………………………………………………...6 | Si réponse 6, précisez |
| CAP11. Quelle est le type de CTA que vous prescrivez le plus souvent ? | AL…………………………….……………………………………1  ASAQ…………………….………………………………………..2  Artemisinin-SP……………………………………………………3  Dihydroartemisinin-piperaquine.....…………………………….4  Artesunate-Mefloquine.....………………...…………………….5  Aucun.………………………………………………………..…...6  Autre.……………………………………………………………...7 | Si réponse 7, précisez |
| CAP12. Quelles sont les raisons des choix des CTA ? | Effets secondaires……………………………………………….1  Expérience personnel……………………………………………2  Autres raisons………………….…………………………………3 |  |
| CAP13. Quels sont les effets secondaires que vous rapportent souvent vos patients recevant les CTA ? | Trouble gastro-intestinal……………………….………………..1  Nausée……………………………………….…...………………2  Vomissements………………………………...………...............3  Asthénie physique……………………………………………….4  Pas d’effet secondaire…………………………………………..5  Autre………………………………………………….……………6 | Si réponse 6, précisez |
| CAP14.quelle est la base de votre prescription de CTA ? | Devant tout cas de fièvre………………….…………………….1  Diagnostic biologique……………………………………………2  Diagnostic clinique……………………………………………….3  Diagnostic biologique + Clinique……………………………….4  Autre……………………………………………………………….5 | Si réponse 5, précisez |
| CAP15. quelles sont les mesures et attitude prises lors de la dispensation des CTA ? | Première dose supervisée par le prestataire………………….1  Répéter la dose initiale si vomissement……………………….2  Informer sur les effets indésirables possibles de l’ASAQ…...3  Cas d’intolérance à l’ASAQ, donner AL ………………..….….4  Organiser une visite de suivi 2 jours après le traitement …...5  Aucune mesure…………………………………………………..6  Autres……………………………………………………………..7 |  |
| CAP16. le prescripteur donne-t-il des conseils au malade ? | Prise d’ASAQ avec boissons sucrées et au cours des repas………………………………………………………………1  L’utilisation de la MILDA………….……………………………………….…2  L’observance du traitement médicamenteux………………..….3  Les signes nécessitant le retour immédiat ………………………...4  L’importance de la visite de suivi………………………………………..5  L’hygiène du milieu de vie et l’assainissement…………………..6  Aucun conseil………………………………………………………………………7  Autres…………………………………………………………………………………8 |  |
| - CAP17.selon vous quels sont les facteurs qui limitent la prescription des CTA ? | Indisponibilité…………………………………….………………1  Payant ………………………………………….…………..…….2  Effets indésirables…………….…………………………………3  Rupture de stock..…………….…………………………………4  Pas de facteurs…………………………………………..………5  Autre…………………………………………….…………..…….6 | Si réponse 6, précisez |
| CAP18. Etes-vous en accord avec l’indication de prescription des CTA en cas de paludisme simple? | Oui totalement…………………………………...……………….1  Oui partiellement………………………………...…………….…2  Non………………………………………………...………………3 | 2,3⇨suivant |
| CAP19. Si partiellement ou non, pourquoi ?, | ………………………………………………...…………………….  ………………………………………………...…………………….  ………………………………………………...…………………….  ………………………………………………...……………………. |  |

Merci

**QUESTIONNAIRE DISPENSATEUR**

*Encerclez les bonnes réponses*

| **Information sur les structures sanitaires** | |
| --- | --- |
| PIS1. Numéro de fiche : | /___/___/___//___/ Automatique |
| PIS2. Numéro du personnel | /_____/ /_____/ /______/ /______ /  Région district structure Numéro |
| PIS3. Nom du district sanitaire | ___ ___ ___ ___ ______ ___ ___ |
| pis4. Nom du service sanitaire | Pharmacie……………………………………...1  Point de vente………………………………….2  Magasin…………………………………………3 |
| PIS4. Nom de la localité | ___ ___ ___ ___ ______ ___ ___ |
| PIS5. Type d’établissement sanitaire | Hôpital régional ……………………………………….….1  Hôpital préfectoral .………………………………...….2  Centre Médical Communal ……………………….…3  Centre de santé urbain………………………………...4  Centre de santé rural…………………………………….5 |
| PIS5. Date de l’interview | /___/___/____/ |

| **Information sur les dispensateurs des médicaments** | | | |
| --- | --- | --- | --- |
| PID1.Quel est l’âge du dispensateur ? | | Âge (en années révolues) …………………………………………… |  |
| PID2. Quel est le genre du dispensateur ? | | Masculin………………………………………..………………….…1  Féminin……………………………..…………………………………2 |  |
| PID3. Niveau de formation du dispensateur ? | | Pharmacien…………………………………………………………..1  Infirmier …………………………………….………………………..2  ATS ………………………………………………..…………………..3  Autre…………………………………………………………………….4 | Si réponse 4, précisez |
| PID4. Au cours des six (6) derniers mois, avez-vous suivi une formation sur le paludisme ? | | Oui………………………………………………………………………..1  Non……………………………………………………………………….2 | 1⇨question  suivante  2⇨ question  PID6 |
| PID5. Quels étaient les thèmes de formation? | | ___ ___ ___ ___ ______ ___ ___ ___ ___  ___ ___ ___ ___ ______ ___ ___ ___ ___ |  |
| PID6. Avez-vous connaissance du protocole national de prise en charge actuel du paludisme ? | | Oui………………………………………………………….……….…..1  Non…………………………………………………………….………..2 |  |
| PID7. Disposez-vous des documents de formation sur la prise en charge du paludisme ? | | Oui…………………………………………………………………..…..1  Non……………………………………………………………………...2 | 1⇨vérifiez et marquer vu. |
| - PID8. La structure a-t-elle reçu une mission de supervision sur le paludisme du niveau supérieur durant les six derniers mois ? | | Oui……………………………………………………………….……….1  Non……………………………………………………………………...2 | 1⇨vérifiez et marquer vu. |
| **Disponibilité et la dispensation des CTA** | | | |
| DDC1. Selon vos observations dans l’exercice, en cas de paludisme simple, que prescrivent les soignants le plus souvent dans votre structure ? | CTA…………………………………………………………………………………..1  Quinine…………………………………………………………………………....2  Autre ………………………………………………………..……………………..3 | |  |
| DDC2. Au cours des six derniers mois, avez-vous eu des ruptures des CTA dans votre structure | Oui…………………………………………………………………………………..1  Non……………………………………………………………………………..…..2 | |  |
| DDC3. Si oui combien de temps a duré cette rupture de CTA ? | /_______/ jours | |  |
| DDC4. Quelles sont les types de CTA présents actuellement dans votre pharmacie /point de vente? | ASAQ………………………………………………………………………………..1  AL……………………………………………………………………………………..2  Autre………………………………………………………………………………..3 | |  |
| DDC5. Quelles sont les formes de CTA présents actuellement dans votre pharmacie /point de vente? | Comprimés……………………………………………………………………….1  Sirops…………………………………………………………………………...….2  Autre………………………………………………………………………………..3 | |  |
| DDC6. Selon vous, quelle est le type de CTA le plus prescrit dans votre structure ? | ASAQ………………………………………………………………………………..1  AL……………………………………………………………………………………..2  Autre………………………………………………………………………………..3 | |  |
| DDC7. Dans votre expérience, avez-vous souvent observé des réticences des patients lors de la délivrance des CTA ? | Oui…………………………………………………………………………………...1  Non…………………………………………………………………………………..2  Rare………………………………………………………………………………….3  Ne sait pas…..……………………………………………………………………4 | |  |
| DDC8. Si oui, Ces réticences ont été observées le plus pour quel type de CTA ? | ASAQ………………………………………………………………………………..1  AL……………………………………………………………………………………..2  Autre………………………………………………………………………………..3 | |  |
| DDC9. Au moment de la dispensation des CTA, quelles sont les conseils que vous donnez ? | Prise d’ASAQ avec boissons sucrées/cours des repas…….…1  L’utilisation de la MILDA……………………………………………..……2  L’observance du traitement médicamenteux.………….…….3  Les signes nécessitant le retour immédiat ………………………4  L’importance de la visite de suivi………………………………………5  L’hygiène du milieu de vie et l’assainissement…..…………...6  Aucun conseil……………………………………………………….………….7  Autres…………………………………………………………..………………….8 | |  |
| DDC10. Vous arrive-t-il d’avoir de la réticence à dispenser un type de CTA | ASAQ…………………………………………………..……………………….…..1  AL……………………………………………….………………………….………..2  Autre…………………………………..……………..……………………….…..3  Non ………………………………….…………….………………………….……4  Ne sait pas………………………………………………………………………..5 | |  |
| DDC11. Si oui, pouvez-vous donner les raisons de cette réticence ? | ………………………………………………………………………………………….  ………………………………………………………………………………………….  …………………………………………………………………………………………. | |  |

Merci

| **Grille pour la Revue documentaire mois de ………………………………………… Structure………..………………………District………………..…….** | | | | | | | | | | | | | | | | | |
| --- | --- | --- | --- | --- | --- | --- | --- | --- | --- | --- | --- | --- | --- | --- | --- | --- | --- |
| **N°** | **Date** | **Age** | **Sexe** | | **Gros-sesse** | **Diagnostic** | | | **Traitement antipaludique** | | | | | **Confirmation**  **biologique** | | **Autre traitement** | |
|  |  |  | M | F | Femme enceinte | Palu simple | Palu grave | \| En plus,noter quelle autre affection \| \| --- \| \| | CTA | | Quinine Cp | Artésunate, arthemeter quinine inj | Autre produit (le quel) | GE +/- | TDR +/- | para ou aspirine | ATB |
|  |  |  |  |  |  |  |  |  | ASAQ | AL |  |  |  |  |  |  |  |
| 1 |  |  |  |  |  |  |  |  |  |  |  |  |  |  |  |  |  |
| 2 |  |  |  |  |  |  |  |  |  |  |  |  |  |  |  |  |  |
| 3 |  |  |  |  |  |  |  |  |  |  |  |  |  |  |  |  |  |
| 4 |  |  |  |  |  |  |  |  |  |  |  |  |  |  |  |  |  |
| 5 |  |  |  |  |  |  |  |  |  |  |  |  |  |  |  |  |  |
| 6 |  |  |  |  |  |  |  |  |  |  |  |  |  |  |  |  |  |
| 7 |  |  |  |  |  |  |  |  |  |  |  |  |  |  |  |  |  |
| 8 |  |  |  |  |  |  |  |  |  |  |  |  |  |  |  |  |  |
| 9 |  |  |  |  |  |  |  |  |  |  |  |  |  |  |  |  |  |
| 10 |  |  |  |  |  |  |  |  |  |  |  |  |  |  |  |  |  |
| 11 |  |  |  |  |  |  |  |  |  |  |  |  |  |  |  |  |  |
| 12 |  |  |  |  |  |  |  |  |  |  |  |  |  |  |  |  |  |
| 13 |  |  |  |  |  |  |  |  |  |  |  |  |  |  |  |  |  |
| 14 |  |  |  |  |  |  |  |  |  |  |  |  |  |  |  |  |  |
| 15 |  |  |  |  |  |  |  |  |  |  |  |  |  |  |  |  |  |
| 16 |  |  |  |  |  |  |  |  |  |  |  |  |  |  |  |  |  |
| 17 |  |  |  |  |  |  |  |  |  |  |  |  |  |  |  |  |  |
| 18 |  |  |  |  |  |  |  |  |  |  |  |  |  |  |  |  |  |
| 19 |  |  |  |  |  |  |  |  |  |  |  |  |  |  |  |  |  |
| 20 |  |  |  |  |  |  |  |  |  |  |  |  |  |  |  |  |  |
| 21 |  |  |  |  |  |  |  |  |  |  |  |  |  |  |  |  |  |
| 22 |  |  |  |  |  |  |  |  |  |  |  |  |  |  |  |  |  |
